# Flower Development in Cassava Is Feminized by Cytokinin, While Proliferation Is Stimulated by Anti-Ethylene and Pruning: Transcriptome Responses

**DOI:** 10.3389/fpls.2021.666266

**Published:** 2021-05-28

**Authors:** Deborah Oluwasanya, Olayemisi Esan, Peter T. Hyde, Peter Kulakow, Tim L. Setter

**Affiliations:** ^1^Section of Soil and Crop Sciences, School of Integrative Plant Science, Cornell University, Ithaca, NY, United States; ^2^Cassava Breeding Unit, International Institute of Tropical Agriculture, Ibadan, Nigeria

**Keywords:** cassava (*Manihot esculenta*), floral development, sex determination, ethylene, transcriptome profiling, RNA-Seq

## Abstract

Cassava, a tropical storage-root crop, is a major source of food security for millions in the tropics. Cassava breeding, however, is hindered by the poor development of flowers and a low ratio of female flowers to male flowers. To advance the understanding of the mechanistic factors regulating cassava flowering, combinations of plant growth regulators (PGRs) and pruning treatments were examined for their effectiveness in improving flower production and fruit set in field conditions. Pruning the fork-type branches, which arise at the shoot apex immediately below newly formed inflorescences, stimulated inflorescence and floral development. The anti-ethylene PGR silver thiosulfate (STS) also increased flower abundance. Both pruning and STS increased flower numbers while having minimal influence on sex ratios. In contrast, the cytokinin benzyladenine (BA) feminized flowers without increasing flower abundance. Combining pruning and STS treatments led to an additive increase in flower abundance; with the addition of BA, over 80% of flowers were females. This three-way treatment combination of pruning+STS+BA also led to an increase in fruit number. Transcriptomic analysis of gene expression in tissues of the apical region and developing inflorescence revealed that the enhancement of flower development by STS+BA was accompanied by downregulation of several genes associated with repression of flowering, including homologs of TEMPRANILLO1 (TEM1), GA receptor GID1b, and ABA signaling genes ABI1 and PP2CA. We conclude that flower-enhancing treatments with pruning, STS, and BA create widespread changes in the network of hormone signaling and regulatory factors beyond ethylene and cytokinin.

## Introduction

Cassava (*Manihot esculenta*) is a perennial tropical plant of the Euphorbiaceae family cultivated as an annual crop for its starchy storage roots (Alene et al., 2018). It constitutes an important source of calories for over 800 million people (Jarvis et al., 2012). It is also a source of starch with expanding potential for industrial applications including a variety of specialty food applications and non-food uses (Li et al., 2017). Continual crop improvement is required to ensure efficient cassava production to meet the growing needs from an increasing population, new uses, and expanded markets, matched with changing environments globally. Cassava improvement has recently received renewed attention with major projects to investigate the potential use of genomic selection in breeding (Wolfe et al., 2017), its source–sink relationships (Sonnewald et al., 2020) and its photosynthetic efficiency (De Souza and Long, 2018). These improvement efforts are geared toward smallholder farmers with an interest in translating cassava end-product quality traits into breeding outcomes (Iragaba et al., 2020). Although cassava can be clonally propagated by stem cuttings, crop improvement *via* breeding requires genetic recombination using sexual crosses between parents from diverse genetic populations that flower in a timely and synchronous manner.

In the Euphorbiaceae family, sexual reproductive structures are referred to as cyathia, which is a modification that has provided this family the advantage of shifting from wind pollination to insect pollination (Horn et al., 2012). A single cassava male cyathium is comprised of multiple reduced stamens (Perera et al., 2013) while a female cyathium possesses a trilocular ovary, meaning that upon pollination, fruits are capable of producing three seeds (Nassar, 1980). Female and male cyathia are separately borne on the same inflorescence. For ease of description, cyathia in this study will be referred to as flowers. Inflorescences and associated flowers are developed from the shoot apical meristem. Following floral initiation at the shoot apex, the two to four buds beneath the inflorescence develop into branches forming a fork. Fork type branches each bear new shoot apical meristems in sympodial growth which after some growth form branches and inflorescences, in turn, at tier 2, tier 3, etc.

There are five bottlenecks with the reproductive development of cassava that challenge breeding. These are: (1) in some genotypes with traits of interest, flowering is late or in some cases there is no flowering at all; (2) premature abortion of inflorescences and flowers before anthesis; (3) disproportionately large number of male flowers and in some cases no female flowers; (4) non-synchronous development of flowers among genotypes; and (5) low probability of fruit development even when flowering and pollination are successful (Ceballos et al., 2004, 2016; Halsey et al., 2008; Adeyemo et al., 2017; Hyde et al., 2020; Souza et al., 2020).

Investigations into the reproductive biology of cassava have led to the development of several potential interventions to improve its reproductive performance: transgenic intervention by overexpressing FLOWERING LOCUS T (Adeyemo et al., 2017; Bull et al., 2017; Odipio et al., 2020), modulating photoperiod and temperature to accelerate flowering time (Adeyemo et al., 2019), application of silver thiosulfate (STS) to enhance flower development and longevity (Hyde et al., 2020), or pruning young subtending branches to alleviate flower abortion. STS is an anti-ethylene plant growth regulator (PGR), which has been used as a foliar spray and was found to be more effective when applied only to the immature shoot apical region (Hyde et al., 2020). So far none of these interventions has focused on increasing the proportion of female flowers. In other members of the Euphorbiaceae family, synthetic cytokinin, benzyladenine (BA), has been used to increase female flower numbers and fruits (Pan and Xu, 2011; Fu et al., 2014; Fröschle et al., 2017; Pineda et al., 2020). Female flowers are critical to cassava breeding; however, flowers on cassava inflorescences are typically only about 10% female, and each female flower produces up to three seeds.

To increase the number of female flowers that develop in cassava plants, we tested the effect of STS, BA, and pruning, singly and combined, on the first flowering event (i.e., flowering at tier 1) under field conditions in Nigeria. We also analyzed the transcriptome of young apical tissues in control plants and in plants receiving either pruning or STS + BA, or the combined set of treatments. We specifically studied the expression pattern of differentially expressed genes relevant to hormone signaling and flower development. Our studies indicated that STS or pruning increase flower numbers over the control, but have no effect on the female-to-male ratios. Conversely, BA was able to almost completely feminize developing flowers, while it did not increase the total number of flowers. Combining BA with STS and pruning provided maximal enhancement in the number of female flowers; this methodology has the potential to assist cassava breeding programs. The observed transcriptome changes in response to these treatments provided further insight into the effects on hormone signaling beyond ethylene and cytokinin and provides insight that will assist future investigations in cassava and other plants.

## Materials and Methods

### Plant Materials

Three genotypes, representing three flowering times and flower prolificacy were used for PGR studies. These were IITA-TMS-IBA980002 (early and profuse), IITA-TMS-IBA30572 (intermediate flowering time and quantity), and TMEB419 (late and minimal flowering). In this article, these genotypes will be referred to as 0002, 30572, and 419, respectively. The genotype 0002 was used to optimize the method of PGR application in 2017. This experiment was conducted between June and December of 2017. Experiments examining the effect of PGRs on female flower development were conducted between June and December of 2018 and 2019.

### Field Conditions

All experiments for phenotyping were conducted under field conditions at the International Institute of Tropical Agriculture (IITA), Oyo State, Ibadan (7.4°N and 3.9°E, 230 m above sea level). The soil was an Alfisol (oxicpaleustalf) (Moormann et al., 1975). The land was tilled and ridged with 1-m spacing; stakes (stem cuttings about 20 cm in length) were planted on top of the ridge. The field in 2017 in Experiment I was previously planted with yam while the field in 2018 and 2019 in Experiment II was previously planted with maize; no extra nutrients or soil amendments were added to the soil. Fields were kept free of weeds with hand weeding.

### Plant Growth Regulators and Method of Application

**Figure 1:**
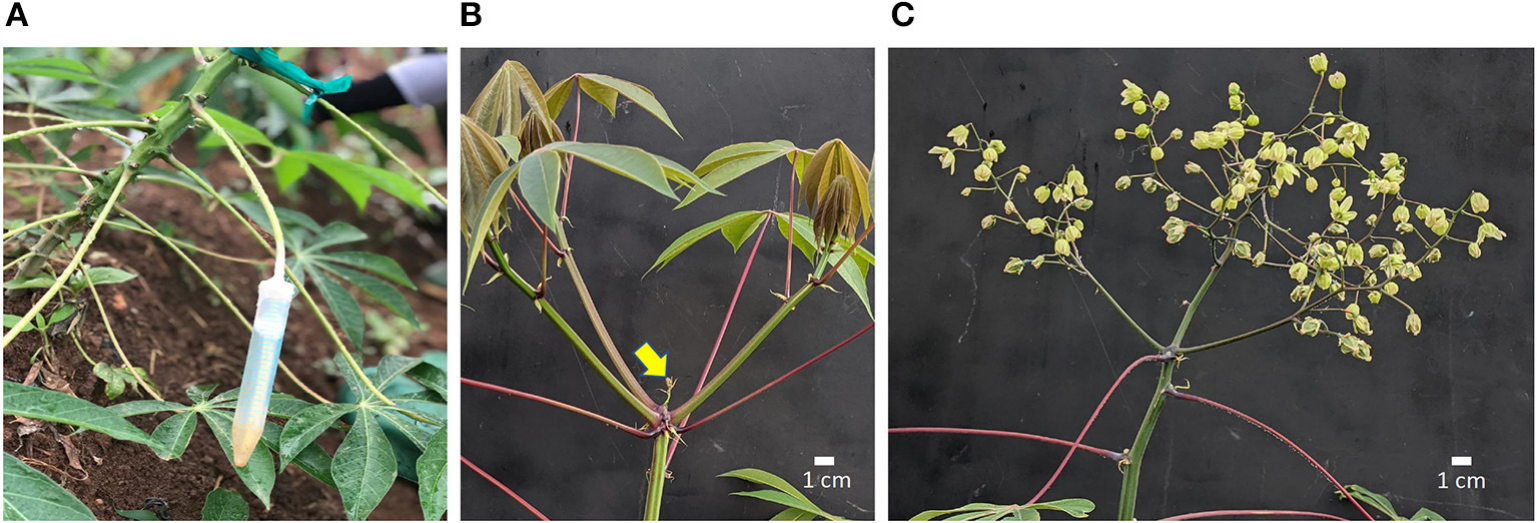
Cassava inflorescence development following treatments. **(A)** Method of PGR application by petiole feeding; **(B)** Control fork-type branching with three developing branches; inflorescence aborting (arrow); **(C)** Inflorescence (flowers and fruit) on a plant that received STS+BA and pruning. Scale bar indicates 1 cm. STS, silver thiosulfate; BA, benzyladenine; PGR, plant growth regulator.

Silver thiosulfate (STS) was prepared by mixing 1 part 0.1 M silver nitrate (AgNO_3_) dropwise with four parts 0.1 M sodium thiosulfate (Na_2_S_2_O_3_), yielding a 20-mM stock solution. The stock solution was diluted with distilled water to the required concentration as specified in each experiment. BA solution was prepared by diluting a 1.9% (w/v) BA stock (MaxCel®, Valent BioSciences Corporation, Libertyville, IL, United States) with distilled water to respective concentrations. PGRs were applied either by spray (about 5 ml) to shoot apex, every 7 days or by the “petiole feeding” method every 14 days. In the petiole feeding method, the leaf blade was removed using a surgical scissors and the petiole was inserted into a 15-ml conical-bottom centrifuge tube (Falcon Brand, Corning, NY, United States) containing 5–10-ml of PGR solution. PGR was taken up *via* the petiole into the xylem from which it was distributed internally to target organs in the apical region (Figure 1A). Petioles were allowed to remain immersed in PGR solution for 72 h after which tubes were removed. On weeks with petiole feeding, spray treatments were applied 24 h after petiole treatments. PGR treatments were initiated 6 weeks after planting.

### Pruning Reproductive Branches

Shoot apexes were inspected weekly using headband magnifier glasses (10 × magnification) to identify forking events. In plants that had forked, young reproductive branches, 2 cm or smaller, were excised carefully without damage to the inflorescence using surgical blades, as previously described (Pineda et al., 2020). Lateral branches which developed about 10 cm below the shoot apex were excised periodically until fruit development. Photos of pruned and unpruned plants are shown in Figure 1.

### Flower Data Collection

The number of male and female flowers and fruits were counted for each plant weekly and recorded using Field Book computer application (Rife and Poland, 2014). Flowers were left uncovered, allowing open pollination by insects. Data were collected for at least 20 weeks. For analysis, the maximum flower or fruit count for each plant over the 20-weeks period was used to represent the response to treatment.

### Plant Growth Regulator Experiment I: Optimization of BA Timing

One hundred plants of IITA-TMS-IBA980002 were grown in a 10 × 10 grid arrangement at a planting distance of 1 × 1 m. The field was divided into 10 replicate plots (blocks) and plants in each plot represented the complete set of treatments in a two-factor design with five BA timing treatments and two STS treatments, creating 10 treatment combinations. At 6 weeks after planting, these 10 PGR treatments were randomly assigned to each plot, with each plant as the experimental unit. BA solution at 0.22 mM was spray-applied at four flower developmental stages; the timing of BA application is summarized in Table 1. For each BA treatment (BA timing), plants were given an STS treatment: either STS or H_2_O *via* petiole feeding. Five milliliters of 8-mM STS was used for the first application, then reduced to 5 ml of 4 mM STS for subsequent applications to limit phytotoxicity.

**Table 1:** Timing of weekly BA applications (spray to shoot apical region) with respect to floral development stages for Experiment I.

**Treatment name**	**Beginning of BA sprays**	**End of BA sprays**
**Flower developmental stage**		
Control	Spray water to shoot apex throughout time frame	
BA_Always	Before flower appearance	End of experiment
BA_Early	Before flower appearance	Flower appearance
BA_Mid	Flower appearance	First flower antheses
BA_Late	21 d after flower appearance	End of experiment

### Plant Growth Regulator Experiment II: PGR and Pruning Effect on Female Flower Development

In 2018 and 2019, experiments were conducted using a split–split–split plot design. The experiment was comprised of six plots (blocks), each of which was split into three subplots with one of the three genotypes in each. Each genotype subplot was split into five PGR treatments (as shown in Table 2) and finally each PGR treatment was split into two pruning treatments—pruned vs. unpruned. In 2018 a total of 1,440 plants were sown, while in 2019, 720 plants were sown. In each subplot with a particular genotype × PGR × pruning combination, there were eight or four replicate plants (subsamples) in 2018 and 2019, respectively. At each level of split, genotypes, PGR treatments, or pruning treatments were randomly assigned. Petiole feeding of STS and BA was with either 10 ml of 2 mM STS (5 ml of 4 mM STS was used in the first month of treatment in 2018), 5 ml of 0.5 mM BA, or a mixture of 5 ml of 2 mM STS and 0.125 mM BA. Spray treatments of BA were applied to the shoot apex with 0.5 mM BA. For the analysis, only plants that had flowers were included in this analysis (plants with no flowers were excluded).

**Table 2:** Summary of PGR treatments for experiment II.

**PGR:**	**BA**	**BA**	**STS**
**Method of delivery:**	**Apical spray**	**Petiole fed**	**Petiole fed**
**Treatment name**			
Control			
BA	×		
BA+BA	×	×	
STS+BA	×		×
STS+BA+BA	×	×	×

### Statistical Analysis

Count data, such as the numbers of total flowers, female flowers, and fruits were analyzed using a negative binomial distribution, as recommended for count data of the sort observed in this study (Lloyd-Smith, 2007; Towers, 2018). In our case, the distributions had additional sources of dispersion relative to that expected in a Poisson distribution. This was expected biologically because counts were zero in many plants, including those plants that aborted inflorescence development before any countable flowers developed (Hyde et al., 2020). This distribution was such that models which would be applied to normal distributions would tend to underestimate dispersion, whereas the negative binomial properly adjusts CIs (Towers, 2018). The negative binomial model (a family of the generalized linear mixed models, GLMM) was therefore applied as described below. Ratios data, such as the proportion of total flowers that were female, were modeled using the binomial mixed model, which does not model fractions directly but models the probability of having females (as success) or males (as failure) while taking into account random effects. Due to similarities of genotypic response to PGR and pruning (different magnitudes of changes but similar trends), the means of genotypes in 2018 and 2019 are reported here.

Models were built using the package Generalized Linear Mixed Models using Template Model Builder (glmmTMB) (Brooks et al., 2017) in R_Core_Team (2017). For Experiment I, fixed effects of the multi-factor model were STS treatment (+STS, –STS), BA timing (Always, Early, Mid, Late, no-BA), and the interaction between STS treatment and BA timing. Plots (complete blocks with all treatment combinations represented) were random effects, which accounted for field spatial variability and accordingly reduced error variance. For Experiment II, fixed effects were PGR treatment (–PGR, BA, BA+BA, STS+BA, and STS+BA+BA; see Table 2), pruning (un-pruned, pruned), the interaction between PGR treatment and pruning, genotype, Year (2018, 2019), representing different growth environments, and Plot, each of which contained the full set of genotypes and treatment combinations. Year was analyzed as a fixed effect while plot as a random effect. In the greenhouse experiment for transcriptomic study, fixed effects were PGR (–PGR, +PGR), pruning (un-pruned, pruned), and the interaction between PGR treatment and pruning. The emmeans package (estimated marginal means, previously called least squares means) (Lenth, 2019) was used for *post-hoc* tests. The multiple comparisons were conducted on the log odds ratio scale using the Tukey method, which makes appropriate adjustments for multiple testing.

### Experiment for Transcriptomic Analyses

IITA-TMS-IBA980002 was grown in a greenhouse at the Guterman Bioclimatic Laboratory (Cornell University, Ithaca, NY, United States) as described by Hyde et al. (2020) and exposed to one of four treatment combinations (a 2 × 2 matrix of treatments): (a) either control or pruned at first inflorescence appearance, and (b) either control or PGR treatment with a combination of petiole-fed STS, and apex-sprayed BA. These were applied to plants at tier 1 of fork branching. Young inflorescence tissue, about 0.25–0.5 cm in length, comprising the shoot apex and some bracts but excluding fork-type branches were harvested from control, pruned, and PGR-treated plants and immediately immersed in liquid N_2_ and transferred to a −80°C freezer for storage. Five biological replicates were analyzed for each treatment combination. Samples were collected 4 days after pruning or at a similar developmental stage in unpruned plants.

Total RNA was extracted from each sample by a modified CTAB protocol. Samples were ground to a fine powder in a mortar and pestle chilled with liquid N_2_; about 0.15 ml of the powder was vigorously mixed for 5 min with 0.4 ml of CTAB extraction buffer [1% [w/v] CTAB detergent, 100 mM Tris-HCl [pH 8.0], 1.4 M NaCl, 20 mM EDTA, and 2% [v/v] 2-mercaptoethanol]; 0.2 ml of chloroform was added and mixed for 1 min, tubes were centrifuged at 14,000 *g* for 10 min and 200 μL of the top layer was removed to a new tube. To these samples was added 700 μL of Guanidine Buffer (4 M guanidine thiocyanate, 10 mM MOPS, pH 6.7) and 500 μL of ethanol (100%). This mixture was applied to silica RNA columns (RNA mini spin column, Epoch Life Science, Missouri City, TX, United States), then washed with 750 μL of each of the following: (1) MOPS-ethanol Buffer [10 mM MOPS-HCl [pH 6.7], 1 mM EDTA, containing 80% [v/v] ethanol], (2) 80% ethanol (twice), and (3) 10 μL RNAase-free water to elute the RNA (twice). The RNA quality was evaluated with a gel system (TapeStation 2200, Agilent Technologies, Santa Clara, CA, United States). The three RNA-seq libraries were prepared from ~500 ng total RNA at the Cornell Genomics facility (http://www.biotech.cornell.edu/brc/genomics-facility) using the Lexogen QuantSeq 3′ mRNA-Seq Library Prep Kit FWD (Greenland, NH, United States).

The libraries were quantified with the intercalating dye QuantiFluor, evenly pooled, and sequenced on one lane of an Illumina NextSeq500 sequencer, single-end 1 × 86 bp, and de-multiplexed based upon six base i7 indices using Illumina bcl2fastq2 software (version 2.18; Illumina, Inc., San Diego, CA, United States). Samples with fewer than 1.5 × 10^5^ demultiplexed reads were excluded from the gene counting analysis. Illumina adapters were removed from the de-multiplexed fastq files using Trimmomatic (version.36) (Bolger et al., 2014). Poly-A tails and poly-G stretches of at least 10 bases in length were then removed keeping reads at least 18 bases in length after trimming. The trimmed reads were aligned to the *Manihot esculenta* genome assembly 520_v7 (https://genome.jgi.doe.gov) using the STAR aligner (version 2.7.0f; Dobin et al., 2012).

Differential gene expression analysis was conducted using the DESeq2 package by Bioconductor (Love et al., 2014). Each transcript was annotated by the best match between *M. esculenta* genome v7 and the Arabidopsis genome as presented at Phytozome13 (Goodstein et al., 2012). Gene ontology and enrichment analyses were carried out using the ShinyGO app (http://bioinformatics.sdstate.edu/go/) (Ge et al., 2020). A combined list of Arabidopsis flowering genes was obtained from the Max Planck Institute (https://www.mpipz.mpg.de/14637/Arabidopsis_flowering_genes) and Flowering Interactive Database (FLOR-ID) (http://www.phytosystems.ulg.ac.be/florid/) (Bouché et al., 2016); a list of hormone signaling genes sourced through the Database for Annotation, Visualization and Integrated Discovery (DAVID) (https://david.ncifcrf.gov/) (Dennis et al., 2003) and a list of Cassava MADS-Box MIKC genes were obtained from iTAK database (http://itak.feilab.net/cgi-bin/itak/db_family_gene_list.cgi?acc=MADS-MIKC&plant=3983) (Zheng et al., 2016). These genes were used to determine the expression pattern of genes by flowering, hormone signaling, and MADS-Box categories, respectively.

## Results

### Plant Growth Regulator Experiment I: Application Method Optimization

In previous studies under greenhouse growth conditions, STS spray treatments increased flower numbers and longevity (Hyde et al., 2020); however, when sprayed onto leaves, cassava foliage sometimes experienced phytotoxic damage. It was possible to substantially decrease the quantity of STS used with similar benefit if the spray was localized to the region surrounding the shoot apical meristem (Hyde et al., 2020). However, when this method was used in preliminary studies in the field, STS effectiveness was not consistent and clear-cut (Setter, personal communication). We, therefore, developed a new method, similar to that reported by Lin et al. (2011), whereby PGRs were fed *via* a cut petiole and delivered to the shoot interior by xylem suction such that phytotoxicity was largely prevented. In the current study, we used this petiole feeding method for STS application in field studies (Figure 1A). Further, we investigated whether BA applied as a spray to the immature tissues affects flower development as a sole treatment or when combined with STS delivered through the petiole.

**Figure 2:**
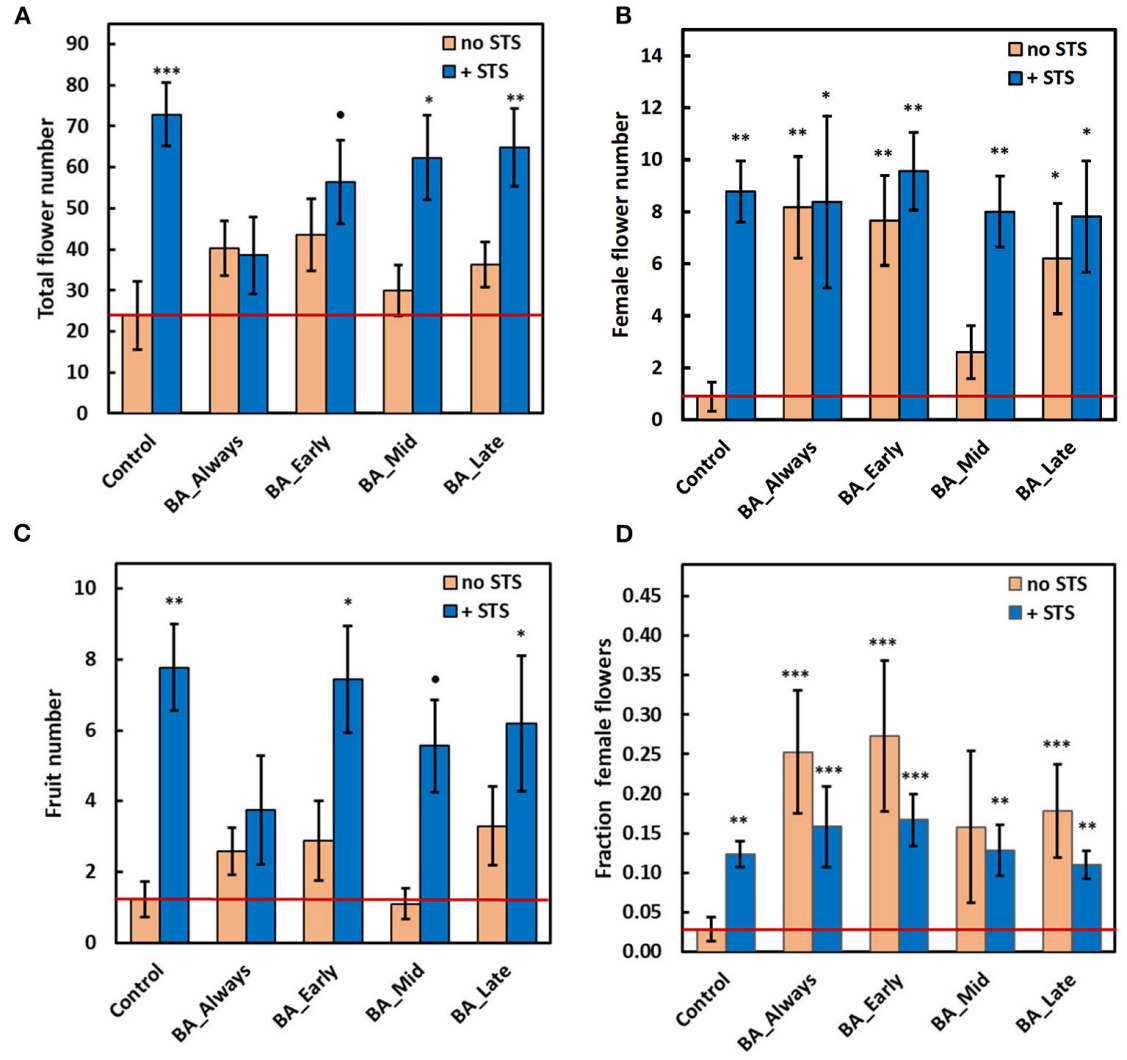
Effect of STS and BA treatments on: **(A)** total flower numbers per plant, **(B)** number of female flowers per plant, **(C)** number of fruit per plant, and **(D)** fraction of female flowers. STS vs. no-STS (H_2_O) treatment is indicated by bar color as shown in the legends. BA treatments were applied throughout floral development (BA Always), or they were initiated at three timings (BA Early, BA Mid, or BA Late). The study was conducted using genotype TMS-I980002 in the field at Ibadan, Nigeria in 2017. See Materials & Methods section for details. Data shown is the mean and SEM of 10 biological replicates; asterisk indicates statistical significance in pairwise comparisons between each treatment and the reference control (no BA, no STS): *P* ≤ 0.10 (•), *P* ≤ 0.05 (*), *P* ≤ 0.01 (**), *P* ≤ 0.001 (***). The value for the reference control is shown as a horizontal red line. STS, silver thiosulfate; BA, benzyladenine.

Compared with the control, without any PGR treatment, STS substantially increased the total number of flowers when applied as a sole treatment, and in combination with BA at early, mid, or late timing (Figure 2A; photo: Figures 1B,C). Statistical results for these findings are also shown in Supplementary Table 1, 2017 worksheet: total flower numbers in plants receiving STS treatments had an overall incidence rate ratio (IRR) of 4.03 and a CI_0.95_ of 2.05–7.94 (*n* = 96). This implies that STS-treated plants were four times more likely to have increased total flower numbers compared with non-STS-treated plants. STS treatment also increased both the number of female flowers (Figure 2B) and the number of fruits (Figure 2C). Corresponding statistics for effect sizes are shown in Supplementary Table 1; female flowers: (IRR = 9.76, CI_0.95_ = 3.01, 31.67), and fruits: (IRR = 5.30, CI_0.95_ = 2.09, 13.41). BA treatments alone did not affect the total flower and fruit numbers (Figures 2A,C), suggesting that the beneficial effect of STS was not dependent on the timing of BA application. However, in comparison with the no-STS/no-BA control, BA applied at all timings except BA-Mid increased the number of female flowers to about the same extent as STS (Figure 2B). These increases were associated with BA-elicited increases in the proportion of total flowers that were female (Figure 2D). Early and continuous BA treatments (with or without STS) were most effective (*P* ≤ 0.001) at increasing the fraction of female flowers relative to the no-PGR controls (BA_early: odds ratio = 6.5, CI_0.95_ = 3.04, 13.88; BA_Always: odds ratio = 7.64, CI_0.95_ = 3.62, 16.11; Supplementary Table 1). Compared with the control, STS alone slightly increased the fraction of flowers that were female (Figure 2D), but the effect of the STS-only treatment was striking in substantially increasing fruit numbers (Figure 2C). In contrast, BA did not affect fruit numbers. The effect sizes of treatments in all treatment combinations, on the response of all traits, are as shown in Supplementary Table 1, worksheet 2017.

### Plant Growth Regulator Experiment II: PGR and Pruning Effect on Flower and Fruit Development

From Experiment I, above, we obtained evidence that STS treatment increases flower and fruit numbers and that spraying BA to the shoot apex increased the proportion of flowers that were female. In Experiment II, we tested two additional factors. First was the potential effect on flowering of pruning branch shoots that arise just below the apical meristem where inflorescences initiate (Pineda et al., 2020). Second was the potential benefit of applying BA *via* petiole feeding rather than only *via* external spray to leaves and/or apical regions. We, therefore, investigated the interaction between continuous BA sprays with: (1) petiole-fed BA; (2) petiole-fed STS; and (3) a mixture of STS and BA (see Materials and methods). We also increased the BA concentration to 0.5mM and studied PGR effects with or without pruning (see Materials and methods). The effect on individual genotypes is shown in Supplementary Table 2 while the combined-genotype averages are presented below.

**Figure 3:**
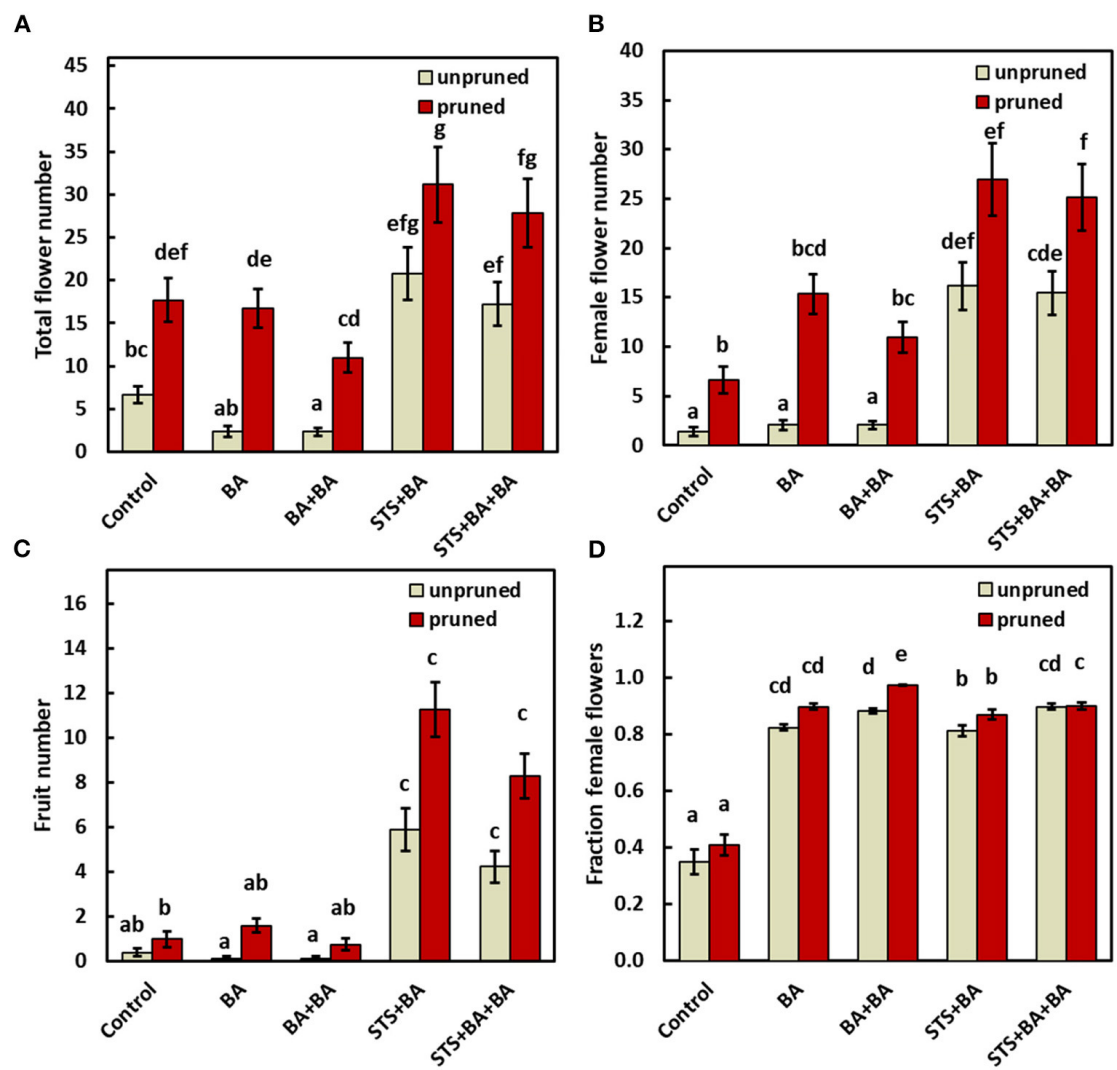
The effect of PGRs and pruning on: **(A)** total flower numbers, **(B)** female flower numbers, **(C)** fruit numbers, and **(D)** proportion of female flowers (reflecting only plants with at least one flower). Data shown is the mean ± SEM of six replicate plots each of which contained the full set of genotype and treatment combinations, and with eight or four subplot plant replicates in 2018 and 2019, respectively. Treatments with different lowercase letters are significantly (*P* < 0.05) different using Tukey's HSD test. Comparisons and letter assignments were based on estimated marginal means (EMMs, least-squares means), as appropriate for statistical comparisons; arithmetic means and SEMs are plotted. PGR, plant growth regulator.

The effects of treatments on all traits measured are shown in Figure 3A. Total flowers were increased by pruning in the absence or presence of BA and STS relative to the control (Figure 3A). Statistics for this finding are also shown in Supplementary Table 1, worksheet 2018–2019 (IRR = 3.07, CI_0.95_ = 2.05, 4.58). In contrast, BA-only did not affect the total number of flowers relative to the control whether applied by spray (BA) or *via* the combination of apical spray and petiole uptake methods (BA+BA). STS-inclusive treatments (i.e., STS+BA and STS+BA+BA) in the absence of pruning significantly (*P* ≤ 0.05) increased total flower numbers relative to the control (Figure 3A; also shown in Supplementary Table 1: STS+BA: IRR = 4.01, CI_0.95_ = 2.74, 5.86; STS+BA+BA: IRR = 3.28, CI_0_._95_ = 2.24, 4.8). Pruning plus STS had the largest number of flowers, with an apparent additive effect of the two treatments (Figure 3A).

The effect of treatments on female flower numbers was similar to the effects on total flower numbers (Figure 3B). BA-only PGR treatments (BA, BA+BA) were not significantly different from the no-PGR treatments without pruning and with pruning (Figure 3B). STS-inclusive treatments (STS+BA, STS+BA+BA) without pruning had significantly (*p* ≤ 0.05) more female flowers than BA-only treatments and the no-PGR unpruned control, but was equivalent to BA+BA and control with pruning. STS-inclusive treatments with pruning had the highest number of female flowers (Figure 3B). Supplementary Table 1, worksheet 2018–2019, shows details on effect sizes.

As with other traits, fruit numbers in the BA-only treatments were not significantly different from control in the absence or presence of pruning (Figure 3C). STS-inclusive treatments, however, increased (*p* ≤ 0.05) fruit numbers relative to the control in both the unpruned and pruned plants.

In the controls, female flowers represented 35–40% of the flowers in both unpruned and pruned plants, with the remainder male flowers (Figure 3D). In contrast, all treatments that included BA were significantly different from the controls and had over 80% females, with or without pruning, and with or without STS.

### Transcriptomics

**Figure 4:**
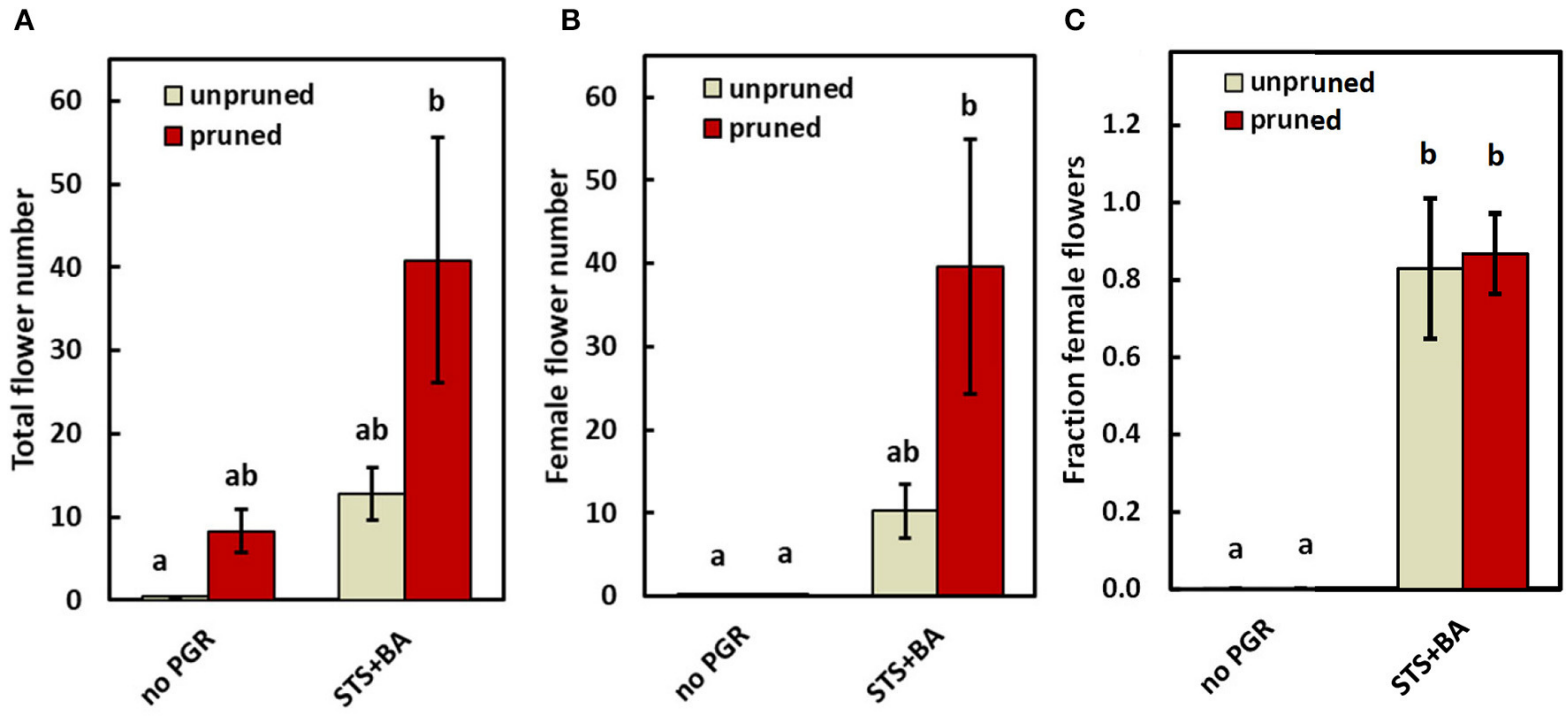
The effect of PGRs (combined STS+BA) and pruning on flower development under greenhouse conditions. **(A)** Total flowers, **(B)** female flowers, and **(C)** proportion of female flowers. Means ± SEM of five biological replicates are shown for the genotype TMS-I980002. Treatments with different lowercase letters are significantly (*P* ≤ 0.05) different using Tukey's HSD test. Comparisons and letter assignments were based on estimated marginal means (EMMs, least-squares means), as appropriate for statistical comparisons; arithmetic means and SEMs are plotted.

To advance our understanding of PGR and pruning effects on flowering regulatory processes, we analyzed gene expression in response to PGR and pruning treatments. For this work, we used treatments that had the largest effect in the field: (a) STS+BA without pruning; (b) STS+BA with pruning, (c) pruning without PGR treatment; and (d) control (no PGRs and no pruning). This study was conducted on the model genotype 0002, in a controlled environment greenhouse. The findings in the growth chamber were consistent with those in the field. Similar to the field study, the controls (unpruned, no PGR) initiated inflorescences, but did not produce any mature flowers, while pruning or STS+BA as sole treatments produced a modest number of flowers (Figure 4A); in the pruning treatment all the flowers were male, but in STS+BA, about 80% were female (Figures 4B,C). Combining STS+BA with pruning increased (*P* ≤ 0.05) total and female flower numbers by almost twofold compared with the PGR- or pruning-only treatments. As with field studies, BA-containing treatments increased the number of female flowers and the proportion of flowers that were female (Figures 4B,C).

**Figure 5:**
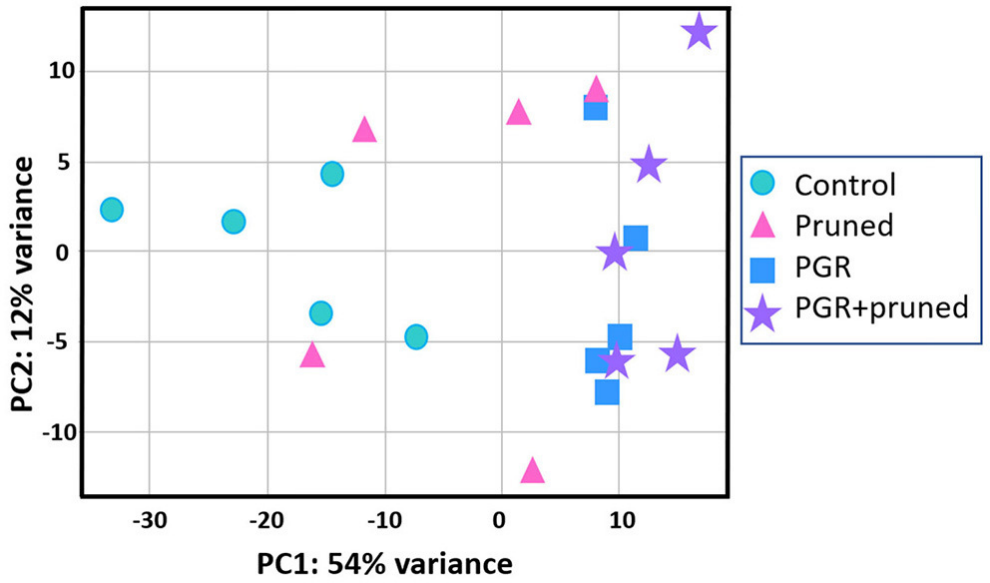
Comparison of DEGs by PCA with respect to control, pruned, PGR and PGR+pruned treatments. Data represent five biological replicates. DEG, differentially expressed genes; PCA, principle components analysis; PGR, plant growth regulator.

**Figure 6:**
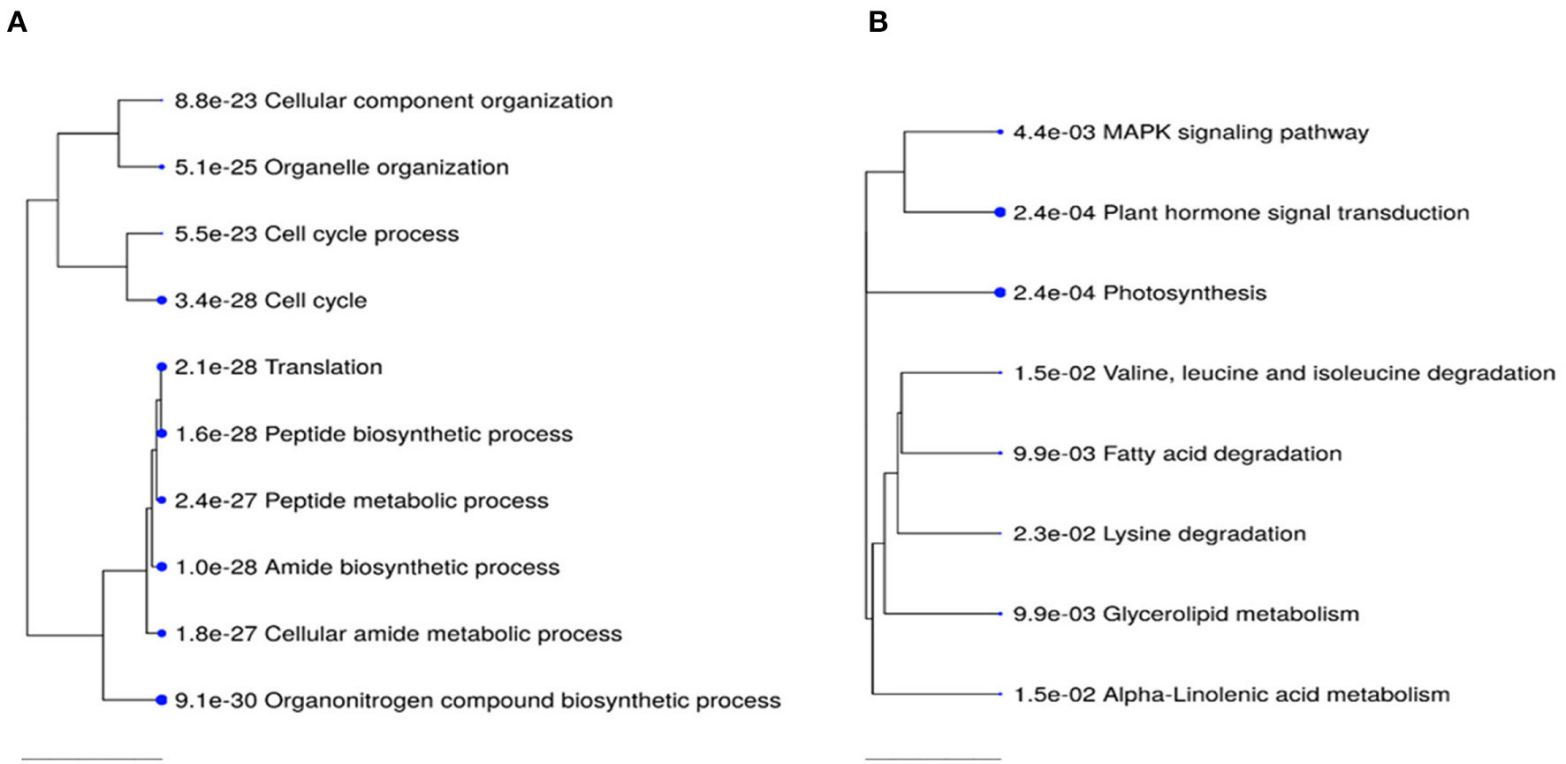
Top 10 KEGG pathway enrichment terms for 2,448 upregulated **(A)** and 2,952 down-regulated **(B)** genes in PGR-treated vs. control comparison. Categories of differentially expressed genes that were significantly enriched relative to the Arabidopsis genome (*P*-values for the lack of enrichment are shown). PGR, plant growth regulator.

Transcriptome analysis was conducted for tissues of the shoot apical region and proceeded in three phases: (i) examining transcriptome changes due to pruning alone (desig*n* = ~pruning), (ii) examining transcriptome changes due to PGR alone (desig*n* = ~PGR), and (iii) examining transcriptome changes due to the combination of pruning and PGR (desig*n* = ~pruning + PGR). This analysis revealed that a larger number of significant (P_adj_ ≤ 0.05) transcriptome changes occurred in response to PGR or PGR+pruning treatments than pruning alone (5,249, 5,440, and 21, respectively) (Supplementary Tables 3–5). Principle component (PC) analysis indicated that expression was not clearly grouped according to pruned vs. unpruned treatments but was clearly grouped according to PGR-treated vs. PGR-untreated samples (Figure 5). This grouping was along the first PC axis, which accounted for 54% of the variance. It appeared from the PC analysis that pruning had an intermediate effect in the positive direction along the first PC whereas PGR had a more substantial effect in the same direction along PC1 axis; the combination of PGR and pruning gave the largest effect in the positive direction of PC1 axis. Analysis of the full model where 5,440 genes were differentially expressed (P_adj_ ≤ 0.05) in response to pruning and PGR indicated that 2,448 genes were upregulated while 2,952 genes were downregulated. Functional analysis revealed that in the PGR-treated vs. controls, PGR-upregulated genes were enriched in pathways involving cell proliferation, cell maintenance, and biosynthetic processes; while PGR-downregulated genes were enriched in pathways involved in plant hormone signal transduction, photosynthesis, and degradation metabolism, among others (Figures 6A,B).

**Figure 7:**
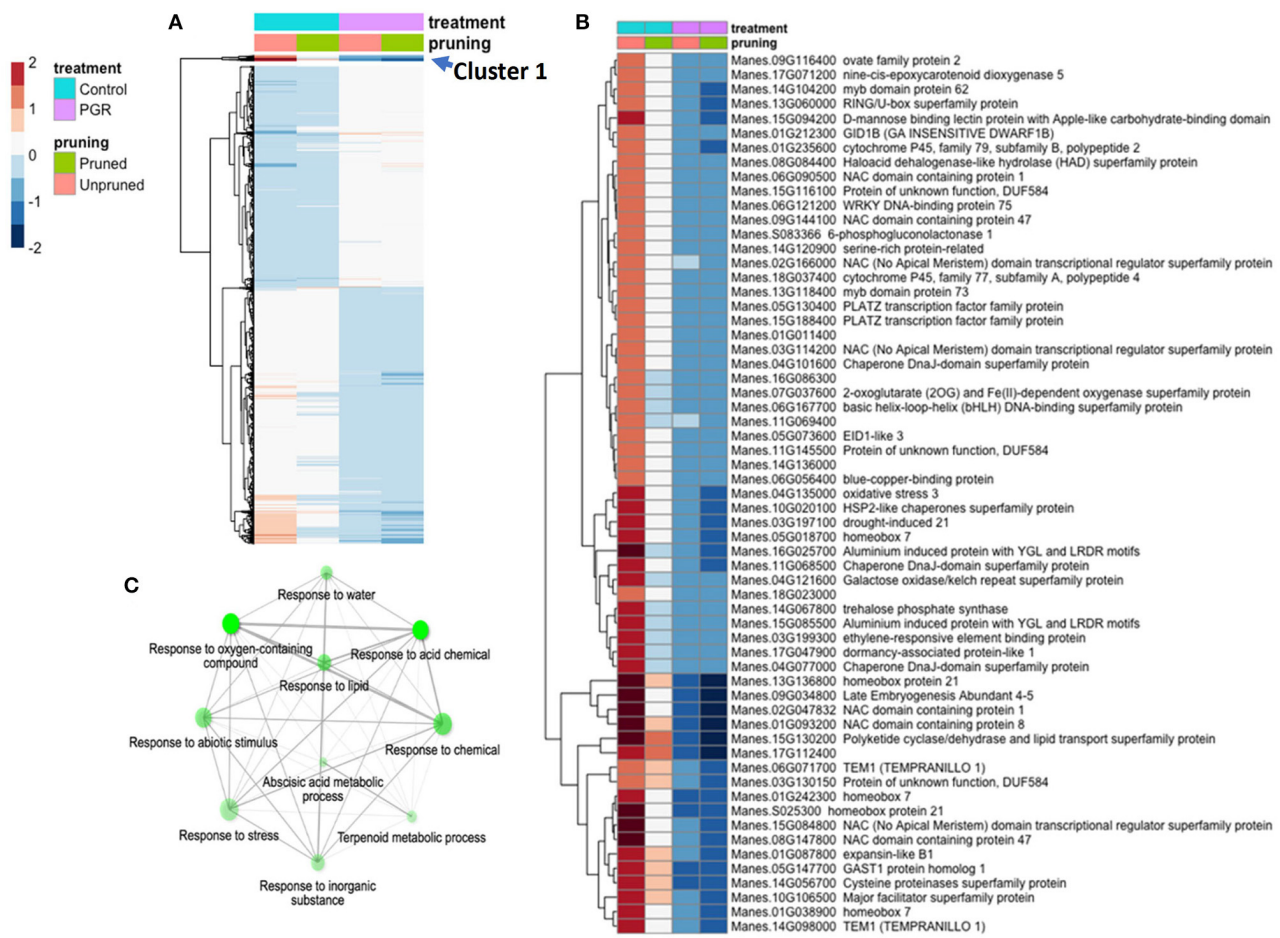
DEGs in response to STS+BA PGRs and pruning. Colors indicate fold change (log_2_ scale shown). **(A)** Full complement of 5,440 differentially expressed genes expressed (P_adj_ ≤ 0.05) (averages of five biological replicates); **(B)** Uppermost slice (Cluster 1); **(C)** Enrichment analysis of the genes in Cluster 1. Color scale indicates log_2_ fold changes. Legend in header indicates color coding of variables. STS, silver thiosulfate; BA, benzyladenine; PGR, plant growth regulator; DEG, differentially expressed genes.

Using the DESeq2 package, we identified genes differentially expressed under the full model (design = ~pruning + PGR) (Figure 7A). Among the four categories of treatment (± PGR × ± pruning), expression was generally grouped according to whether the category was +PGR vs. –PGR. Many genes in plants that received the pruning treatment but were not treated with PGR were intermediate between the control and the PGR-treated plants. By inspection of the fold-expression heatmap, we selected a cluster of genes with exceptionally high fold changes compared with expression profiles of other genes. Genes in this cluster (Cluster 1, Figure 7A) had high expression in the control, low expression in PGR-treated plants, and intermediate expression with pruning only. Enrichment analysis indicated that this cluster, consisting of 61 genes, was enriched with genes involved in abscisic acid metabolism and response, terpenoid metabolism, abiotic stress response, and response to chemicals (Figures 7B,C). This cluster suggests that regulation associated with the treatments that stimulate flowering decreases the expression of stress-associated genes, which were expressed at relatively higher levels in the control apical region.

**Figure 8:**
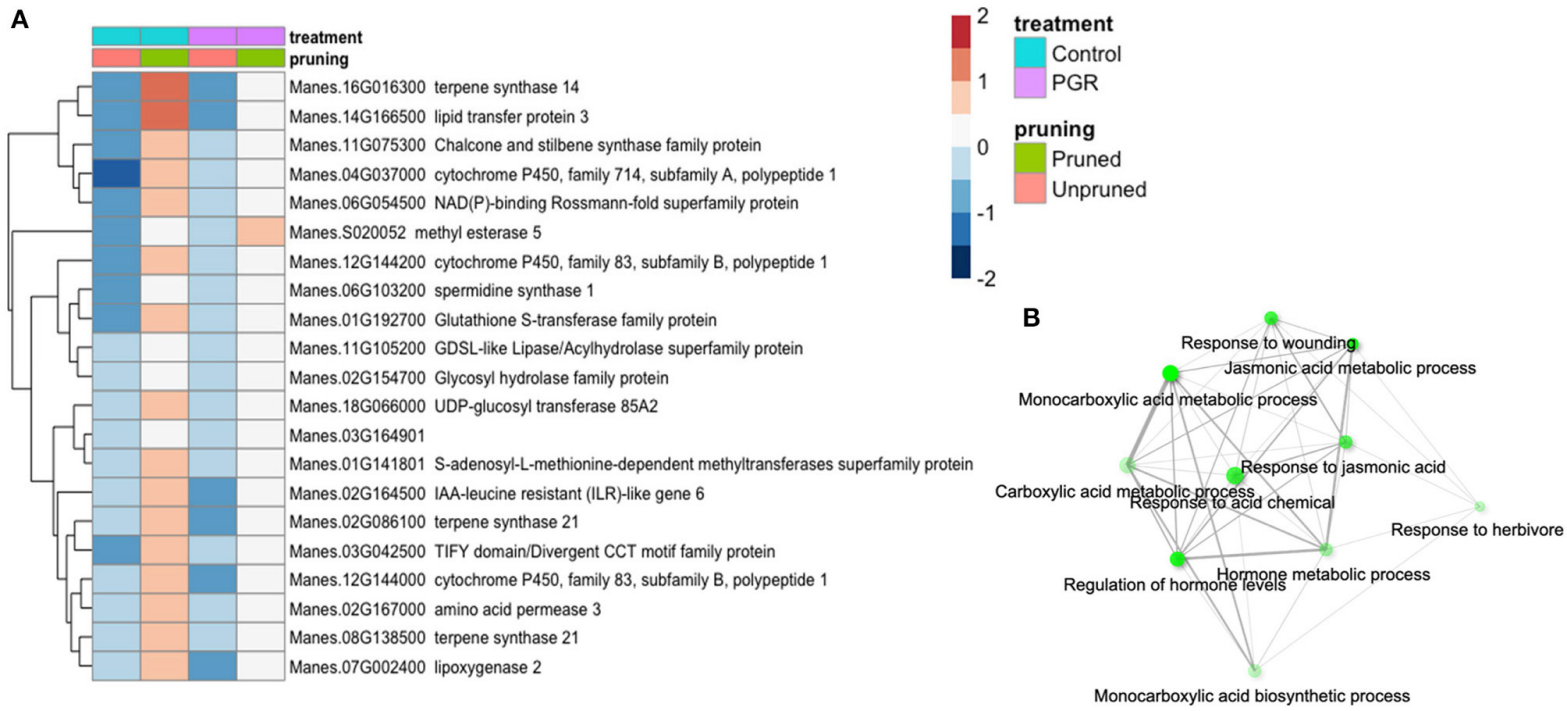
**(A)** Differentially expressed genes identified as statistically significant (P_adj_ ≤ 0.05) (averages of five biological replicates) in response to pruning only, but not PGRs. **(B)** Enrichment analysis of pruning-responsive genes. Colors indicate fold change (log_2_ scale shown). Legend in header indicates color coding of variables.

Twenty-one genes were differentially expressed (P_adj_ ≤ 0.05) in response to pruning as the sole treatment (Figure 8A). This set was enriched in genes involved in response to wounding, herbivory, and jasmonic acid signaling (Figure 8B). Also upregulated in pruning were genes involved in terpene, lipid, and hormone metabolic pathways. Pruning increased expression of these genes in the presence or absence of PGR treatments. Given that the tissues for this analysis were harvested 4 d after pruning, it is not surprising that metabolic and signaling factors involved in wounding response were expressed abundantly. Both pruning and PGR treatment (STS+BA) were effective as sole treatments in increasing flower numbers, so we evaluated the genes that both treatments either increased or decreased relative to the control. For this assessment, a threshold differential of |0.5| (log_2_) was used to identify genes from among the genes for which differential expression was found (P_adj_ ≤ 0.05). This yielded a set of 63 genes that were upregulated in both PGR and pruning treatments and 224 genes that were downregulated in both treatments, relative to the control. These genes were subjected to enrichment analysis (Ge et al., 2020), however, they did not reveal enrichment categories with clearly relevant functions. The two categories with the strongest evidence for enrichment among upregulated genes were S-adenosylmethionine metabolic process (three genes, P = 2.6 × 10^−4^) and aromatic amino acid biosynthesis (four genes, *P* = 5.2 × 10^−4^). The two categories with the strongest evidence for enrichment among downregulated genes were response to oxygen-containing compound (49 genes, *P* = 8.3 × 10^−15^) and response to abiotic stimulus (42 genes, *P* = 8 × 10^−14^).

#### Hormone Signaling Genes

**Figure 9:**
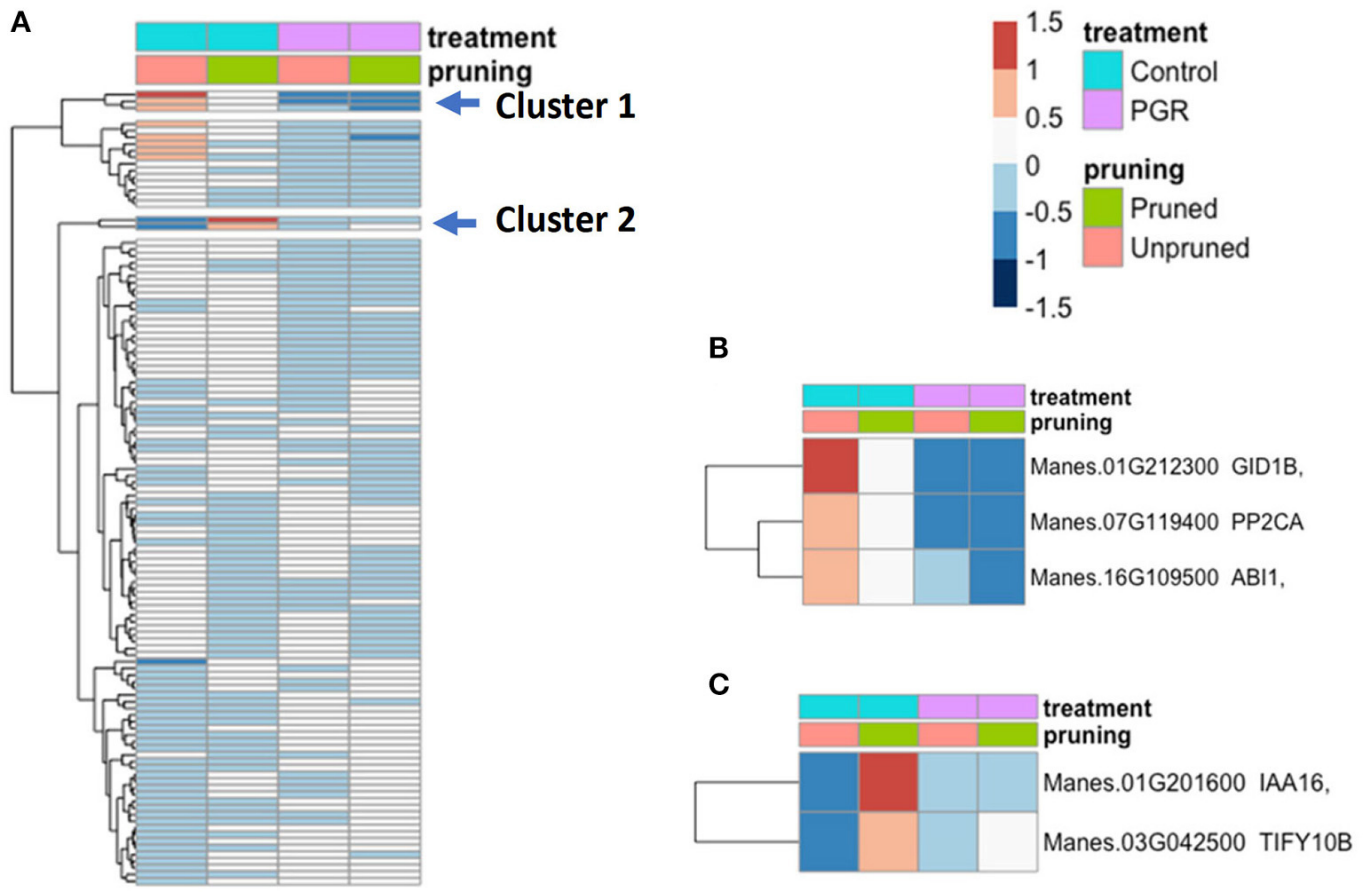
**(A)** Differentially expressed hormone-signaling genes in response to PGR and pruning. Clusters of highly affected genes are indicated with arrows. Clusters 1 and 2 are shown in detail in **(B,C)**, respectively. Averages of five biological replicates. Color scale indicates log_2_ fold changes. Legend in header indicates color coding of variables. PGR, plant growth regulator.

We examined the expression profile of 115 hormone signal-transduction genes (Dennis et al., 2003; Zheng et al., 2016) that were differentially expressed (P_adj_ ≤ 0.05) in our samples (Figure 9; expression data for the full set of hormone signaling genes is available in Supplementary Figure 1). While many genes differed only modestly between treatments, there was a cluster of genes with relatively high expression in the control and substantially lower expression in the PGR treatments (Cluster 1). This cluster included a GA signaling gene (GID1b), and two repressors of ABA signaling (ABI1 and PP2CA) (Figure 9B). We also identified an auxin response gene (IAA16), and a JA response gene (TIFY10B) that were expressed at a low level in the control, and moderately low expression in PGR treatments, but high expression in the pruned without PGR treatment (Figure 9C). Thus, the expression data indicated that pruning and PGR treatments comprising the anti-ethylene treatment STS and cytokinin treatment BA influenced the expression of genes in hormone signaling pathways other than cytokinin and ethylene pathways, suggesting these hormones have a considerable breadth of impact in the networks of hormone signaling.

#### Flowering Genes

**Figure 10:**
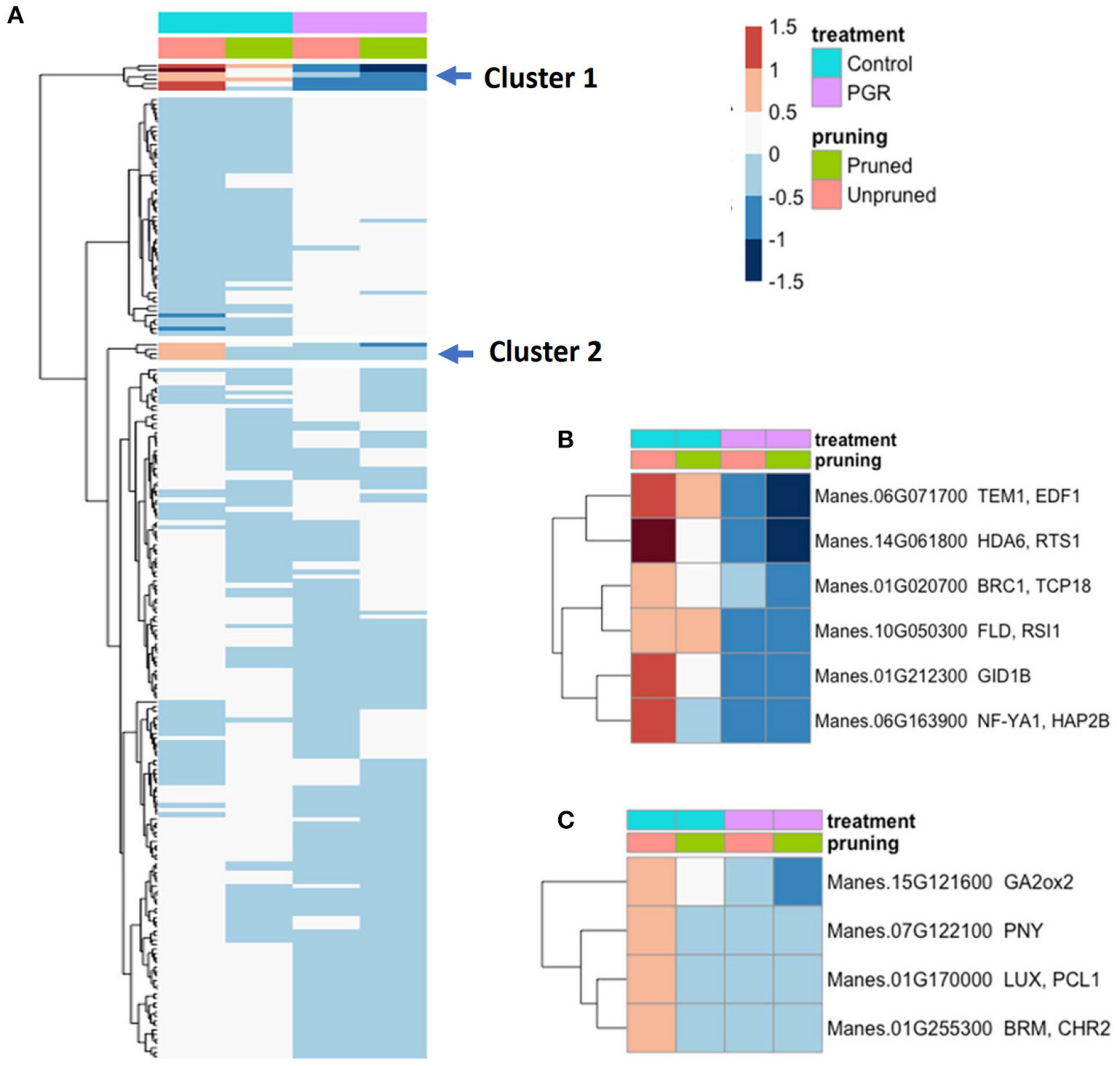
**(A)** Expression of 217 differentially expressed flowering genes in response to PGRs and pruning. Clusters of highly affected genes are indicated with arrows. Clusters 1 and 2 are shown in detail in **(B,C)**, respectively. Averages of five biological replicates. Color scale indicates log_2_ fold changes. Legend in header indicates color coding of variables. PGR, plant growth regulator.

Among genes known to be involved in the timing of floral initiation and flower development (Bouché et al., 2016), we identified 217 genes that were differentially expressed (P_adj_ ≤ 0.05) in response to our experimental treatments (Figure 10A). From the two clusters with the largest fold changes, two members of the GA flowering pathway (GID1b, GA2ox2), and TEM1, a known flowering repressor, had the highest fold change with relatively high expression in controls, and low expression in the PGR treatments (Figures 10B,C). Expression profiles of flowering genes organized by known flowering pathways are presented in Supplementary Figure 2. Other flowering genes with large fold changes with respect to treatments were HDA6, BRC1, FLD, NF-YA1, PNY, LUX, and BRM (Figures 10B,C).

Thirty of the 37 putative MADS-Box MIKC transcription factors (TFs) in cassava (http://itak.feilab.net/cgi-bin/itak/db_family_gene_list.cgi?acc=MADS-MIKC&plant=3983) (Zheng et al., 2016) were differentially expressed (P_adj_ ≤ 0.05) in response to PGR treatment or pruning (Supplementary Figure 3). The fold changes in expression levels of MADS-Box TFs were notably smaller than for the gene groups identified above. We, however, focused our attention on the flower organ development genes, with respect to the ABCDE TFs (Supplementary Figure 4). Generally, the A, B, C, and E class genes had lower expression levels under PGR treatments while the D class genes had higher expression under PGR treatments. In contrast, an AP3 gene—Manes 0.02G100400 (B class) and SEP3—Manes 0.13G009600 (E class) responded to pruning with decreased expression with or without PGR treatment. Among these MADS-Box factors, many responded in a PGR-specific manner in which pruning without PGR was about the same as the control.

## Discussion

### Silver Thiosulfate Increased Total Flower and Fruit Numbers but Did Not Affect Female-to-Male Ratio

A previous study demonstrated that when cassava was grown in a greenhouse environment, STS, an anti-ethylene PGR, maintained flower production and prevented premature flower senescence, leading to a larger number of total flowers (Hyde et al., 2020). The present study demonstrated that STS has similar effects in field environments. STS alone increased the total number of flowers by over two-fold relative to the no-STS controls (Figure 2). In other plant species, ethylene has been shown to hasten the senescence of mature flowers, and anti-ethylene treatments such as STS have been developed to increase flower longevity (Serek et al., 2006). The current findings and those of Hyde et al. (2020) indicate that in cassava, an added effect of anti-ethylene treatment is to prevent the arrested development of newly emerged inflorescences and immature flowers. The current study also indicated that STS treatment proportionally increased the number of female and male flowers, such that female-to-male ratios were relatively unchanged.

In addition to the effects on flowering, effects on flowering, the current study also showed that STS increased fruit numbers (Figures 2, 3), such that the fraction of female flowers that set fruit increased from 0.17 in the absence of STS to 0.31 in treatments that included STS. While ethylene is widely recognized as playing a role in fruit ripening (Pech et al., 2018), it has also been shown to affect the early stages of fruit development and fruit set. In pea (*Pisum sativum*) and Arabidopsis, failure to develop fruit in the absence of pollination has been associated with ovary senescence arising from increased ethylene biosynthesis in ovaries (Orzáez and Granell, 1997; Carbonell-Bejerano et al., 2011). In Zucchini squash (*Cucurbita pepo*), blocking ethylene perception by STS extended ovule lifespan and increased the chance of developing fruit either by pollination or by parthenocarpy in response to gibberellins (Martínez et al., 2013). In contrast to the masculinizing effect of STS on female flowers of *Cannabis* (Ram and Sett, 1982) and *Cucurbita* (Den Nijs and Visser, 1980), STS alone had no effect on the female-to-male fraction (Figure 2D), perhaps because cassava already produces a high fraction of male flowers under natural conditions.

### Pruning and STS had Similar Effects

Pruning of young developing branches just below a newly initiated inflorescence has recently been shown to improve flower development in cassava and increase the total number of flowers, fruits, and seeds (Pineda et al., 2020). Hence, pruning can have similar effects to those reported for STS (Hyde et al., 2020). In addition, we observed that while both pruning and STS increased the number of total flowers, and increased female flowers as a consequence of the overall increase in flower numbers, these two treatments did not affect the ratio of female-to-male flowers (Figures 3, 4). The extent to which STS and pruning treatments elicit their effects by similar mechanisms was explored with our transcriptomics data (below) and is open for further investigation.

### Benzyladenine Increased Female-to-Male Ratio but had No Effect on Total Flower and Fruit Numbers

Our studies indicated that application of cytokinin *via* localized spray to the shoot apical region increased the female-to-male ratio while maintaining the number of total flowers (Figures 2, 3). This effect has also been observed in response to foliar spray in other members of Euphorbiaceae including *Jatropha curcas* (Pan and Xu, 2011; Chen et al., 2014; Pan et al., 2014) and *Plukenetia volubilis* (Fu et al., 2014), which are more prolific than cassava in total flower production. The BA effect on feminization was not dependent on the co-application of STS or pruning treatments which increase total flower production (Figure 3). The feminizing effect of BA on flower development was dose-dependent in *Jatropha curcas* (Chen et al., 2014), in agreement with the present study of cassava with BA as a sole treatment. By increasing the concentration of BA between Experiments I and II, the percentage of female flowers increased from about 12% in the untreated control, to about 30% with 0.22 mM BA, to over 80% with 0.5mM BA (Figures 2, 3). Our studies are also in agreement with studies that have shown that BA is more effective at producing female flowers when treatments are begun at an early stage in flower development (Fröschle et al., 2017; Luo et al., 2020).

In contrast to the effect of BA in increasing the proportion of flowers that were female, BA had no effect on the total number of flowers and fruits (Figures 2, 3). This differs from studies in other members of Euphorbiaceae in which BA treatments also resulted in an increase in the total number of flowers and fruits (Pan and Xu, 2011; Chen et al., 2014; Fu et al., 2014; Pan et al., 2014). On the other hand, when either STS or pruning was combined with BA, the female-to-male ratio was maintained in a larger population of flowers.

A few studies of Euphorbiaceae-family plants have shown that pruning (Pineda et al., 2020), STS (Hyde et al., 2020), or BA (Fu et al., 2014) affect flower development when applied separately, but until the current study, these treatments were not applied together. The current study showed that combining these three factors substantially improved reproductive development in cassava. While STS and pruning acted additively to increase the total number of flowers, BA increased the female-to-male ratio of flowers. This in turn led to greater fruit development than in each of the factors applied singly (Figures 3, 4).

### Regulation of Gene Expression With Pruning Combined With STS+BA

In tissues of the young inflorescence region, the largest number of significantly (P_adj_ ≤ 0.05) affected genes with substantial response to STS+BA treatments were those downregulated relative to their expression in the control. Among the cluster of such genes with the largest fold change (Figure 7), pathway enrichment analysis indicated that pathways related to stress were over-represented compared with their frequency in the genome. These and other examples of signaling by PGRs in our study will be further discussed below. Genes responding to pruning as the main treatment on the other hand were upregulated relative to treatments with no pruning and were enriched in processes generally related to wounding (Figure 8) (Reymond et al., 2000). Given that pruning involves excision of young fork-type branches, it may be perceived as a type of wounding by the plant. Although the tissues used for RNA extraction did not include those directly cut by pruning, a mobile signal such as jasmonate may have elicited transcriptional responses in surrounding tissues. These genes, however, had lowered expression when pruning was combined with STS+BA, the treatment combination which produced the largest number of female flowers and fruits. This suggests that increased expression of wounding-related genes may not be necessary for the benefit derived from pruning in flower and fruit improvement, and indeed, lowering them with STS+BA might be beneficial.

### Hormone Signaling: PGR Treatments Modulate GA and ABA Signaling

Cassava homologs of the Arabidopsis transcripts Gibberellin-Insensitive Dwarf1b (GID1b) and Gibberellin 2-oxidase2 (GA2ox2) were downregulated by PGR treatments (Figures 9, 10). In some species, such as tea (*Camellia sinensis*) and *Magnolia* x *soulangeana*, GID genes are expressed at elevated levels when floral induction takes place (Jiang et al., 2020; Liu et al., 2020). GA2 oxidases, on the other hand, inactivate bioactive gibberellins (Thomas et al., 1999). GA2ox2 has also been shown to be involved in the negative regulation of flowering time in combination with other GA2 oxidases (Rieu et al., 2008). The downregulation of GA2ox2 and GID1b genes by STS+BA suggests that this treatment modulates GA signaling at both biosynthetic (i.e., GA2ox2) and perception (i.e., GID1b) levels. Consistent with this role, studies of tree peony (*Paeonia suffruticosa*) showed that GID and GA2ox2 homologs had higher expression in the control buds than in buds stimulated to form flowers, and this response was interpreted to act in feedback regulation of GA synthesis (Guan et al., 2019).

In addition to affecting GA-related genes, BA+STS treatment downregulated a cassava homolog of ABSCISIC ACID INSENSITIVE1 (ABI1; PP2C family protein) and ABSCISIC ACID HYPERSENSITIVE GERMINATION1 (AHG1, also known as PP2CA) (Figure 9). In contrast, BA application to inflorescences of *Jatropha curcas* upregulated a PP2C homolog (Pan et al., 2014; Gangwar et al., 2018). However, in soybean (*Glycine max*) floral buds, STS decreased expression of PP2C and ethylene-generating treatment increased PP2C (Cheng et al., 2013), consistent with our findings in cassava. These genes encode protein phosphatases that repress ABA signaling and are sometimes expressed in circumstances where ABA signaling is being modulated (Kuhn et al., 2006; Nishimura et al., 2007; Lynch et al., 2012). Details into the role of and in the increase in sensitivity to ABA combined with a decrease in GA perception in cassava merits further study, especially because GA-ABA antagonism is known to be involved in sex specification in ferns (Menéndez et al., 2006; McAdam et al., 2016).

### IAA16 and TIFY10b Respond to Pruning

The expression levels of cassava homologs of INDOLEACETIC ACID-INDUCED PROTEIN16 (IAA16, repressor of auxin responses) and TIFY10b (transcription factors with a conserved amino acid domain—TIF[F/Y]XG; regulator of jasmonate responses) (Vanholme et al., 2007) were highest in the pruning treatment, without PGRs (Figure 9). This suggests a positive relationship between these hormone-signaling genes and pruning (Korasick et al., 2014). IAA is known to be synthesized in young leaves of lateral buds and subsequently transported in the polar auxin stream; pruning lateral buds disrupts this flux and improves fruit yield (Xu et al., 2020, and references cited therein). These findings on pruning-related expression patterns of IAA16 and TIFY10b have identified possible leads for future investigation.

### Plant Growth Regulator and Pruning Treatments Modulate Flowering Genes

Our transcriptome study was not designed to separate the influences of BA on feminization and STS on flower proliferation, so the findings apply collectively to floral development, production, and longevity. Among the genes with the largest fold changes in the current study were a group of flowering-related genes that were downregulated by STS+BA treatment (Figure 10). These included genes that in Arabidopsis have roles as positive flowering effectors, such as HISTONE DEACETYLASE6 (HDA6) (Chen et al., 2010), and NUCLEAR FACTOR Y, SUBUNIT A1 (NF–YA1) (Mu et al., 2013), and negative flowering effectors, BRANCHED1 (BRC1) (González-Grandío et al., 2017), and BRAHMA1 (BRM1, an ATP dependent chromatin remodeler) (Peirats-Llobet et al., 2016). In addition to their roles in flowering (Bouché et al., 2016), these genes also possess functions related to ABA response, and all had similar expression patterns: they were downregulated by BA+STS treatment.

Among the most strongly downregulated genes in response to BA+STS was a homolog of TEMPRANILLO1 (TEM1), a repressor of floral development. TEM1 is a member of the RAV family of transcription factors that are involved in suppressing flowering by downregulating FT, and also have roles in abiotic stress responses (Matías-Hernández et al., 2014). Our finding that pruning and BA+STS downregulate TEM1, suggests that TEM1 has roles in floral development beyond juvenility (Sgamma et al., 2014), possibly providing a link between repressed GA and ethylene signaling (Matías-Hernández et al., 2014). This is reasonable given that the mode of action of STS is silver ion binding to the ethylene receptor and thereby inhibiting ethylene perception (Veen, 1983). In addition, the RAV gene family to which TEM1 belongs has recently been shown in rice (*Oryza sativa*) to play a role in carpel development (Osnato et al., 2020), consistent with the suggested involvement of TEM1 in cassava female flower development. Furthermore, there is evidence that TEM1 can function as a downstream effector of ethylene responses and also suppress the biosynthesis of bioactive GA (Osnato et al., 2012). These findings for TEM1 and other flowering- and hormone-pathway genes advance our understanding of the regulatory systems involved and provides a start toward identifying regulatory pathways that might be candidates for future interventions.

## Conclusion

This study showed that combined treatment with pruning, plus anti-ethylene (STS) and cytokinin (BA) substantially improved cassava female flower and fruit development, and has potential value in promoting reproductive development in cassava breeding programs. It also distinguished treatment effects: BA feminizes floral development and increases the fraction of female flowers, whereas pruning and STS treatment maintain inflorescence development such that more flowers and fruits are produced. Transcript profiling of tissues in the inflorescence region indicated that treatments affected signaling components in multiple hormone and flowering regulatory pathways, including those involving auxin, GA, ethylene, jasmonate, and ABA, with the predominate response in these pathways a downregulation relative to controls. The finding that pruning and BA+STS downregulates TEM1 and other factors that have been found to inhibit flowering advances our understanding of potential regulatory systems that are involved in cassava flowering and are modified by pruning and PGR treatments.

## Data Availability Statement

The datasets presented in this study can be found in online repositories and are available from Cassavabase, https://www.cassavabase.org, a website maintained by the Next Generation (NEXTGEN) Cassava Breeding Project. The data may be accessed at ftp://ftp.cassavabase.org/manuscripts/Oluwasanya_et_al_2021 and are available in accordance with Creative Commons Attribution (CC-BY) license.

## Author Contributions

DO, PK, and TS obtained funding. PK supervised fieldwork in Nigeria. TS supervised greenhouse and lab work in the United States. DO, OE, and PH conducted the field and greenhouse experiments. DO analyzed the data. DO and TS wrote the manuscript. All authors contributed to the article and approved the submitted version.

## Conflict of Interest

The authors declare that the research was conducted in the absence of any commercial or financial relationships that could be construed as a potential conflict of interest.

## Supplementary Material

Supplementary Figures 1–4 Differentially expressed genes (P_adj_ ≤ 0.05) in response to pruning and PGR treatments.

Supplementary Table 1 Worksheet 2017: Results of negative binomial and binomial mixed model regression. Incidence rate ratio (IRR) and odds ratio with 95% CIs for the effects of STS treatment and timing of BA application (Experiment I) on total flowers, numbers of female flowers, fruits, and female-to-male ratios. *n* = 96 (dead plants excluded). Worksheet 2018–2019: Results of negative binomial and binomial mixed model regression. IRR and odds ratio with 95 % CIs for the effects of Pruning, BA and STS inclusive treatments on total flowers, numbers of female flowers, fruits, and female-to-male ratios. *n* = 2,115 (dead plants excluded).

Supplementary Table 2 Mean values and standard deviation for genotypes IITA-TMS-IBA980002, IITA-TMS-IBA30572, and TMEB419 in Experiment II (2018–2019). Shown are the effects of PGRs and pruning on: total flower numbers, female flower numbers, fruit numbers, and proportion of female flowers (reflecting only plants with at least one flower).

Supplementary Table 3 Differentially expressed genes (DEGs) with respect to control, pruned, PGR, and PGR+pruned treatments. DESeq full model.

Supplementary Table 4 Differentially expressed genes (DEGs) with respect to control and PGR treatments. DESeq pgr vs. control.

Supplementary Table 5 Differentially expressed genes (DEGs) with respect to control and pruned treatments. DESeq pruned vs. unpruned.
