# Supplementary material for: Flower Development in Cassava Is Feminized by Cytokinin, While Proliferation Is Stimulated by Anti-Ethylene and Pruning: Transcriptome Responses
Source: Front Plant Sci. 2021 May 28;12:666266. doi: 10.3389/fpls.2021.666266 (PMC8194492; doi:10.3389/fpls.2021.666266)

Supplementary Figure 1A. Genes in hormonal pathways that were differentially expressed (p_{adj} <0.05) in response to pruning or PGR. Colored scale indicates fold change of log(2) expression.

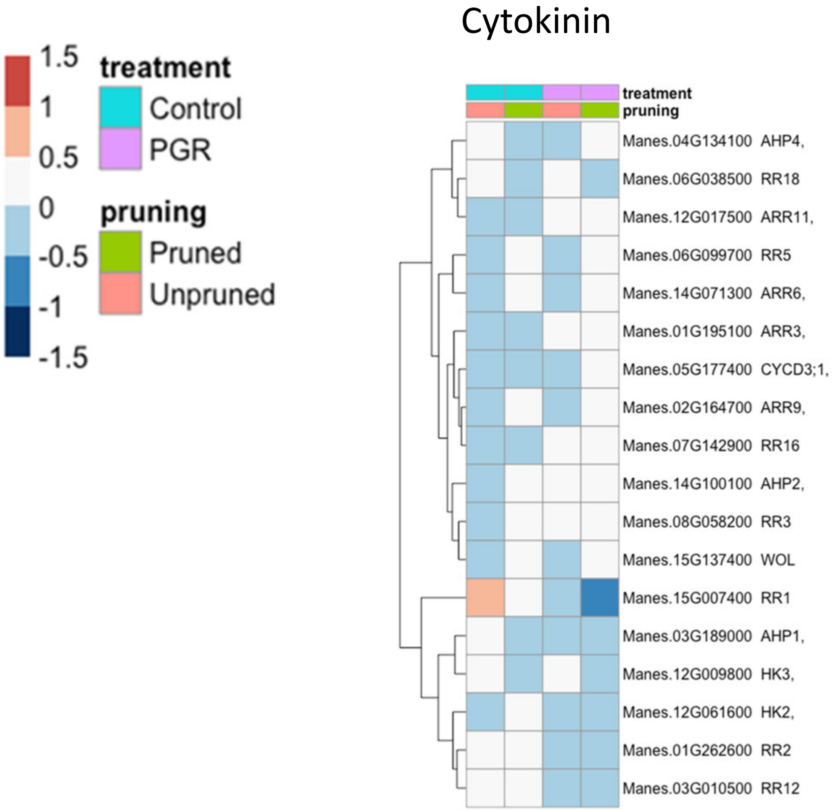

Supplementary Figure 1B. Genes in hormonal pathways that were differentially expressed (p_{adj} <0.05) in response to pruning or PGR. Colored scale indicates fold change of log(2) expression.

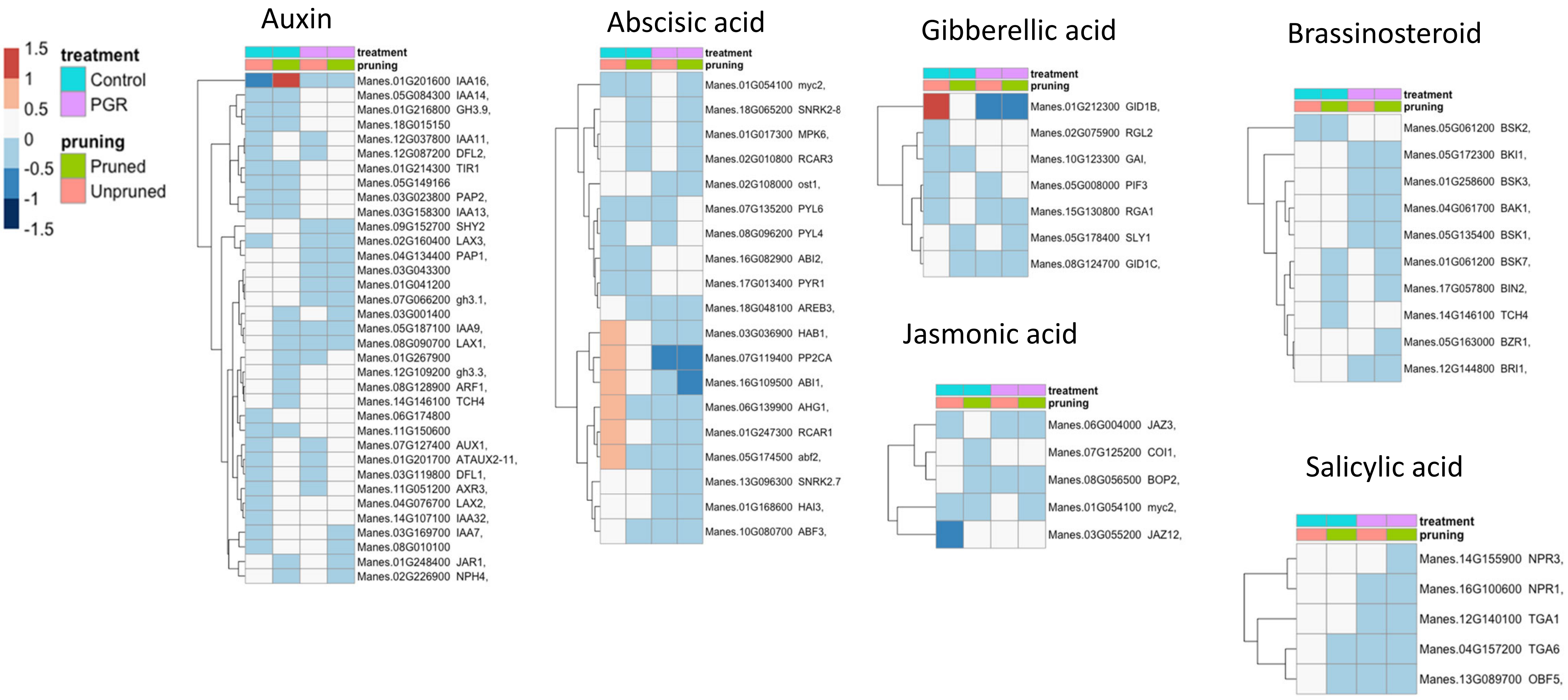

Supplementary Figure 2A. Flowering genes of various signalling pathways differentially expressed (p_{adj} <0.05) in response to pruning and PGRs. Colored scale indicates fold change of log(2) expression.

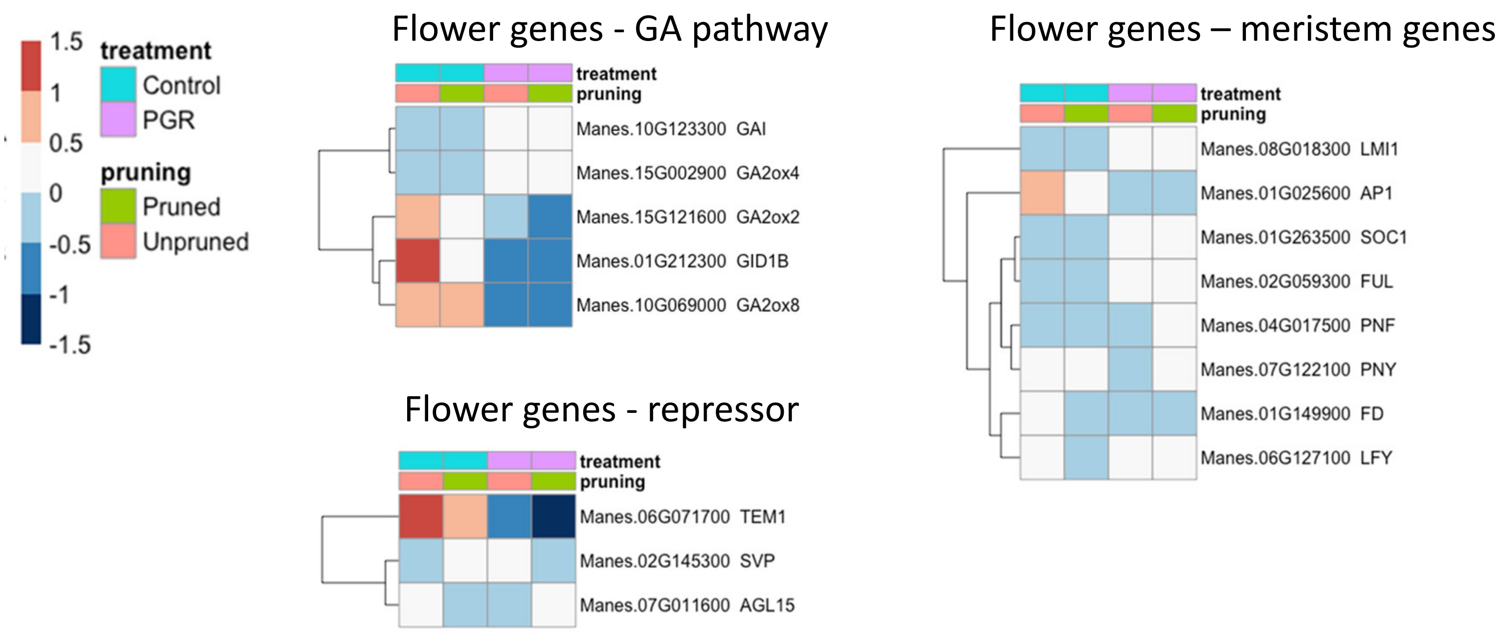

Supplementary Figure 2B. Flowering genes of various signalling pathways differentially expressed (p_{adj} <0.05) in response to pruning and PGRs. Colored scale indicates fold change of log(2) expression.

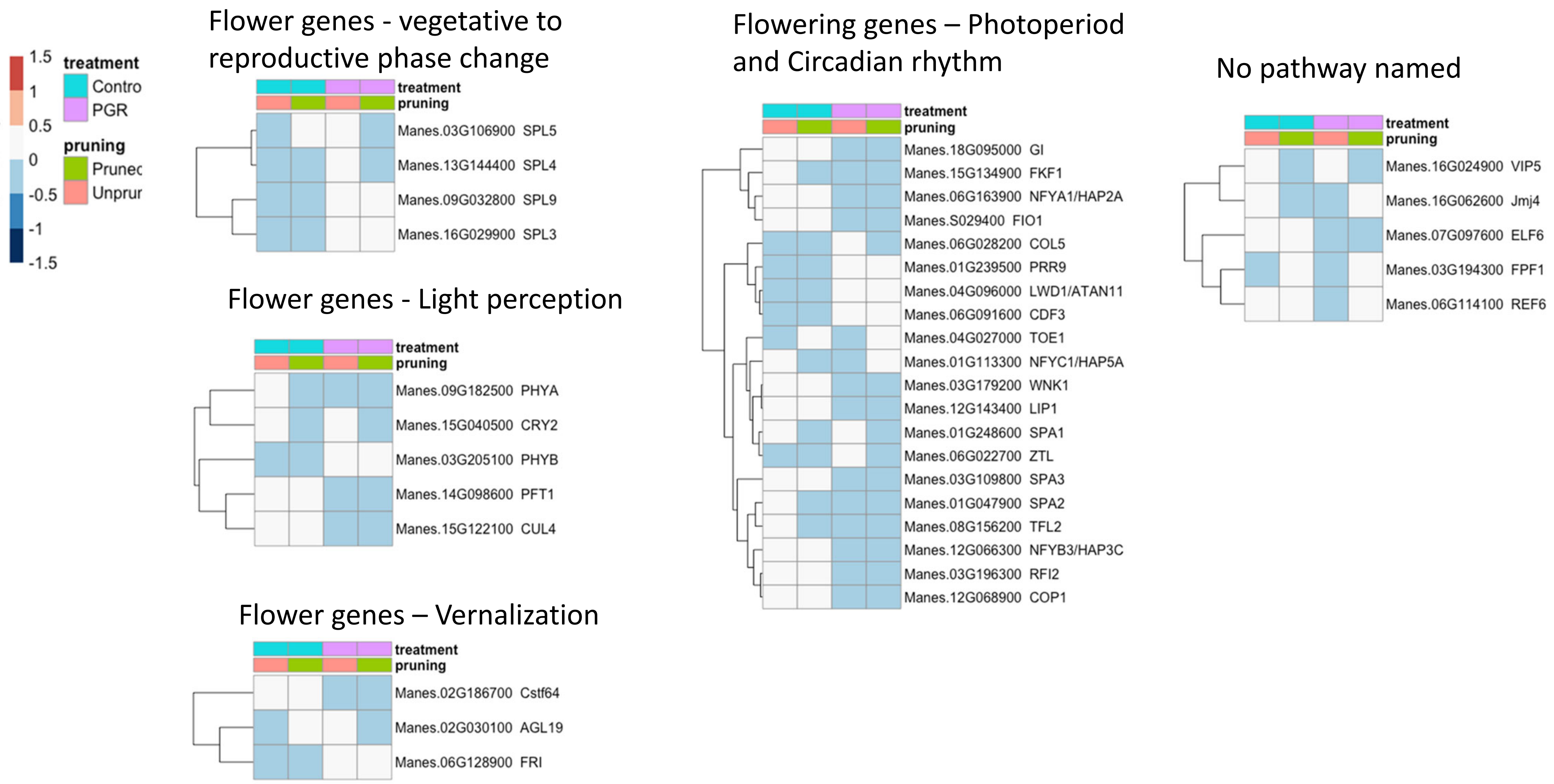

Supplementary Figure 3. MADS Box MIKC genes differentially expressed (p_{adj} <0.05) in response to pruning and PGRs. Colored scale indicates fold change of log(2) expression.

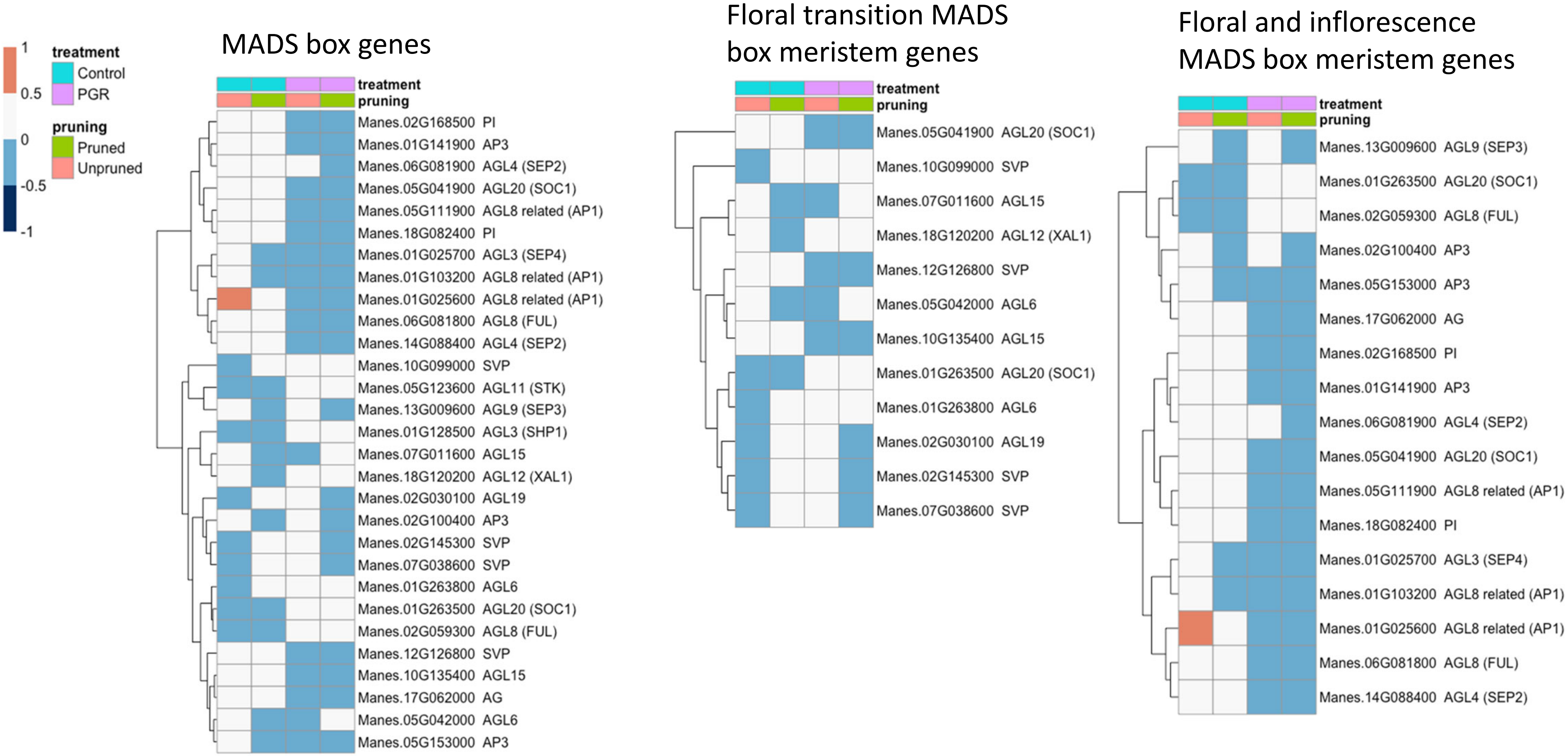

Supplementary Figure 4. Flowering regulatory genes that were differentially expressed ($P_{adj} \leq 0.05$) in response to PGR and pruning treatments. Shown are genes in each class of MADS-Box transcription factors in the ABCDE model for flower developmental regulation. Colored scale indicates fold change of log(2) expression.

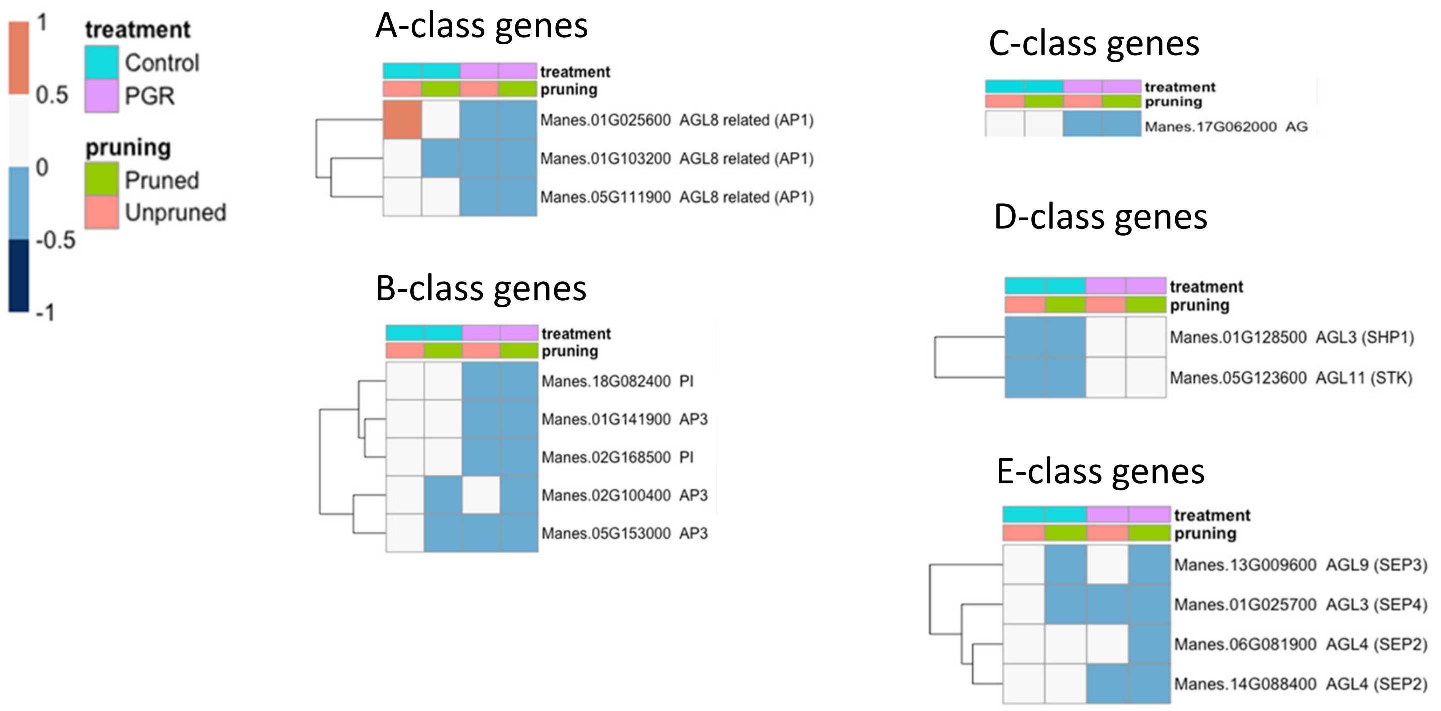

Supplement: Supplementary Figures 1–4 — Differentially expressed genes (Padj ≤ 0.05) in response to pruning and PGR treatments. [file Data_Sheet_1.pdf]
